# Impact of Self-Esteem and Self-Perceived Body Image on the Acceptance of Cosmetic Surgery

**DOI:** 10.7759/cureus.18825

**Published:** 2021-10-16

**Authors:** Hussain A Al Ghadeer, Maisa A AlAlwan, Mariyyah A AlAmer, Fatimah J Alali, Ghadeer A Alkhars, Shahad A Alabdrabulrida, Hasan R Al Shabaan, Adeeb M Buhlaigah, Mohmmed A AlHewishel, Hussain A Alabdrabalnabi

**Affiliations:** 1 Paediatrics, Maternity and Children Hospital, Al-Ahsa, SAU; 2 Plastic Surgery, King Fahad Hospital-Hofuf, Al-Ahsa, SAU; 3 Plastic Surgery, King Faisal University, Al-Ahsa, SAU; 4 Urology, King Fahad Hospital-Hofuf, Al-Ahsa, SAU; 5 Orthopaedics, King Faisal University, Al-Ahsa, SAU; 6 General Surgery, King Fahad Hospital-Hofuf, Al-Ahsa, SAU

**Keywords:** cosmetic surgery, body appreciation, plastic surgery, self-esteem, saudi arabia

## Abstract

Backboard

Cosmetic surgery is the preservation, rebuilding, or improvement of the physical appearance of an individual through surgical and non-surgical methods. In the last few years, an increase in the number of cosmetic procedures was noticed worldwide. This increase suggests due to multifactorial changes in people’s attitudes towards cosmetic surgery and concern about their physical appearance. This study aims to assess the impact of self-esteem and self-perceived body image on the acceptance of cosmetic surgery and other related factors in the Eastern province of Saudi Arabia.

Material and methods

This was a cross-sectional study carried out in the Eastern region of Saudi Arabia. The study was conducted between May and August 2021. A self-administrated questionnaire was distributed to all the participants who are attending plastic surgery clinics and online through social media. Three valid and reliable scales were used [Acceptance of Cosmetic Surgery Scale (ACSS), Body Appreciation Scale (BAS), Rosenberg Self-Esteem Scale (RSE)] to assess the relationship between these variables and other factors. The data were analyzed by using two-tailed tests. P-value less than 0.05 was statistically significant. Correlation analysis was done by using the Pearson correlation coefficient (r).

Results

A total of 1008 participants were included in the study with a response rate of 67%. Participant's ages ranged from 18 to 54 years with a mean age of 34.7 ± 11.2 years old. The study participants showed an average level of acceptance with a mean score % of 55.4% comparing to body appreciation; it was 74.2% higher with a more than average level of self-esteem, 24.7 out of 40 points for self-esteem with a mean score of 61.8%. Participants with a history of cosmetic surgery had significantly higher acceptance score than who did not (mean score of 72.6 compared to 57.1; P=0.001). Male participants had better body appreciation than females (mean score of 50.2 vs. 47.6, respectively; P=0.013). A weak positive correlation with no significance was found between participants’ self-esteem and their acceptance of cosmetic surgery.

Conclusion

A better understanding of the acceptance of cosmetic surgery from a different cultural perspective and other related factors including social, psychological, and self-esteem are crucial for the plastic surgeon to ensure patient satisfaction.

## Introduction

Cosmetic surgery is the preservation, restoration, or improvement of a person's physical appearance which includes changes in any part of the body. It is done by surgical and medical techniques in the presence or the absence of any diseases, injuries, or congenital defects [1,2]. Cosmetic surgeries were done previously for people who need reconstruction of some congenital lesions. Later on, the purpose of applying cosmetic surgery was increased and changed to be more focused on beauty purposes and reversing the effects of aging. This can be due to the high impact on beauty by people nowadays [3]. Worldwide, there has been a huge increase in the prevalence of cosmetic surgeries over the past 10 years [2]. This increase in the prevalence of cosmetic surgery indicates that there is a significant change in people's perception and acceptance of cosmetic surgery [4]. According to the International Society of Aesthetic Plastic Surgery (ISAPS), the total surgical and nonsurgical procedures increased by 7.4% in 2019 compared to 2018 statistics. Patients who seek cosmetic surgeries vary in gender and age. However, the prevalent groups seeking cosmetic surgery are females aged 35-50 years old. Globally, the most common surgical procedures in women were breast augmentation, liposuction, and eyelid surgery, in contrast to men, gynecomastia, eyelid surgery, and liposuction was the most common. The most popular nonsurgical procedures for both genders are botulinum toxin, hyaluronic acid, and hair removal. However, among the top 30 countries with the highest rates of plastic surgery in the world, Saudi Arabia ranks 29 [5]. The prevalence of Saudi females who seek cosmetic surgery is increased dramatically over the last few years [6]. There are lots of factors that make a person decide between seeking cosmetic surgery and consequently increase the rate of cosmetic procedures. These factors can be summarized into three main categories: the characteristics of the patient, the general surrounding of the patient, and the huge development and accessibility of the medical field [7]. The first category: the characteristics of the patient which represents the demographical data of the patient such as age, gender, economic status, and educational level. There was a study conducted in Iran which showed that these demographical data affect the decision to make a cosmetic surgery [8]. The physical status, BMI of the patient, psychological status, and the personality of the patient which represents stress, body image, self-esteem, body dysmorphic disorder, and confirmatory can affect the decision of seeking cosmetic surgery. Another Iranian study showed that self-esteem, body image dissatisfaction, and conformity are effective in the acceptance of cosmetic surgery [9]. The second category: the general surrounding of the patient which is characterized by the culture of the country and the social media. A systemic review was done to emphasize the huge effect of cultural differences and social media on seeking a cosmetic procedure [10]. Also, there was a study conducted in Al-Ahsa, Saudi Arabia suggests that social media has a high impact on body appreciation and deciding to perform cosmetic surgery [11]. The third category: the huge development and accessibility of the medical field which is characterized by the huge increase of cosmetic centers and plastic surgeons all over the world. Many types of research have been done to find the relationship between these factors and seeking cosmetic surgery. In this research, it is focused on the impact of self-esteem and self-perceived body image on the acceptance of cosmetic surgery and other associated factors among the citizen of the Eastern province, Saudi Arabia.

## Materials and methods

Aims

This study aims to evaluate the impact of self-esteem and self-perceived image on the acceptance of cosmetic surgery and body satisfaction among Saudi citizens in the Eastern region.

Study design and participates

A cross-sectional-based study was conducted in the period between May and August 2021. The study population was Saudi citizens who live in the eastern province aged 18 years old and older.

Data collection instrument and procedures

A questionnaire was fulfilled by whom attending plastic surgery clinic and distributed online through social media. Verbal and written consent was obtained from the participant after explaining the purpose of this study. Study objectives guided a self-administered questionnaire. The survey covered four sections through several validated scales used for different specific purposes. The first section included demographic data, such as sex, age, marital status, educational, and family income. The scales used included the Acceptance of Cosmetic Surgery Scale (ACSS), Body Appreciation Scale (BAS), and Rosenberg Self-Esteem (RSE) Scale. The data were collected by using validated questionnaires modified for Arabic speakers.

Demographics

All participants provided their gender, age, education, job, and marital status, income, and whether they underwent cosmetic surgery or not.

ACSS

To measure the acceptance of cosmetic surgery in this study, ACSS was used as a validation scale that was modified for Arabic speakers for the measurement of attitudes toward cosmetic surgery. It is a 15 based on a 7-points scale (1 = strongly disagree, 7 = strongly agree). ACSS runs in three subscales with five items each. The Intrapersonal scale has five items, which assesses the degree to which an individual believes that cosmetic surgery will add to their self-image. The Social subscale has five items, which measures whether an individual would undergo cosmetic surgery for social reasons. Finally, the Consider subscale has five items, which measure the likelihood that an individual will consider having cosmetic surgery. ACSS score ranges from 15 to 105. The higher scores on the sub-scales and the total scale indicate more of a positive attitude toward cosmetic surgery [12,13].

BAS

The BAS is a 13 on 5-points scale (1 = never, 2 = seldom, 3 = sometimes, 4 = often, 5 = always). It is used to assess body appreciation. Participants are asked to select the five options on statements such as "I feel that my body has at least some good qualities" and "My self-worth is independent of my body shape or weight." The average is calculated to obtain an overall body appreciation score. The higher the score, the more body appreciation is scored. The BAS has been shown to have good discriminant, construct, and incremental validities. Among Arabs countries, the BAS has been shown to have good discriminant, constructive, and incremental validities [14,15].

RSE scale

The RSE scale has 10 items on a four-point scale used widely to assess self-worth. The questionnaire was answered on Likert's four points (0 = strongly disagree, 3 = strongly agree). This scale has five negative items required to be reversed before the calculation of the total score. A higher score indicated higher SE. This scale is reliable and valid and used in different studies including the Arabic version [16-18].

Data analysis

After data were extracted, it was revised, coded, and fed to statistical software IBM SPSS version 22 (SPSS, Inc. Chicago, IL). All statistical analysis was done using two-tailed tests. P-value less than 0.05 was statistically significant. For different scales, the overall score was obtained after summing up all discrete items scores after reversing scores for negative statements (in self-esteem scale); then, score percent of maximum was calculated through dividing the overall score by the maximum score for each scale. Descriptive analysis based on frequency and percent distribution was done for all variables including demographic data, and cosmetic surgery history. Mean score and score % with standard deviation were calculated for self-esteem, cosmetic surgery acceptance, and body appreciation scales. The distribution of measured scales by participant's socio-demographic data was assessed using ANOVA test and independent t-test for significance. Correlation analysis was done to assess the relationship between participant's acceptance of cosmetic surgery, body appreciation, and their self-esteem using the Pearson correlation coefficient (r).

## Results

The survey was administered to 1500 eligible participants; 1008 responded (67% response rate). Participant's ages ranged from 18 to 54 years with a mean age of 34.7 ± 11.2 years old. The majority of respondents were females (73.5%; 741) and 768 (76.2%) were married. As for educational level, 860 (85.3%) participants were university graduated. As for work, 339 (33.6%) were not employed while 383 (38%) were non-healthcare workers and 286 (28.4%) were health care workers. A monthly income of 5000-10,000 SR was reported among 319 (31.6%) while 405 (40.2%) had a monthly income of 10,000-20,000 SR. Exactly, 160 (15.9%) participants had chronic health problems (Table 1).

**Table 1:** Sociodemographic data of study participants

Socio-demographic data	No	%
Age in years		
18–24	242	24.0%
25–34	275	27.3%
35–45	317	31.4%
>45	174	17.3%
Gender		
Male	267	26.5%
Female	741	73.5%
Marital status		
Single	202	20.0%
Married	768	76.2%
Divorced/widow	38	3.8%
Educational level		
Intermediate	16	1.6%
Secondary	132	13.1%
University	860	85.3%
Work		
Not employed	339	33.6%
Health care field	286	28.4%
Non-health care field	383	38.0%
Monthly income		
<5000 SR	111	11.0%
5000–10,000 SR	319	31.6%
10,000–20,000 SR	405	40.2%
>20,000 SR	173	17.2%
Have a chronic health problem		
Yes	160	15.9%
No	848	84.1%

**Figure 1:**
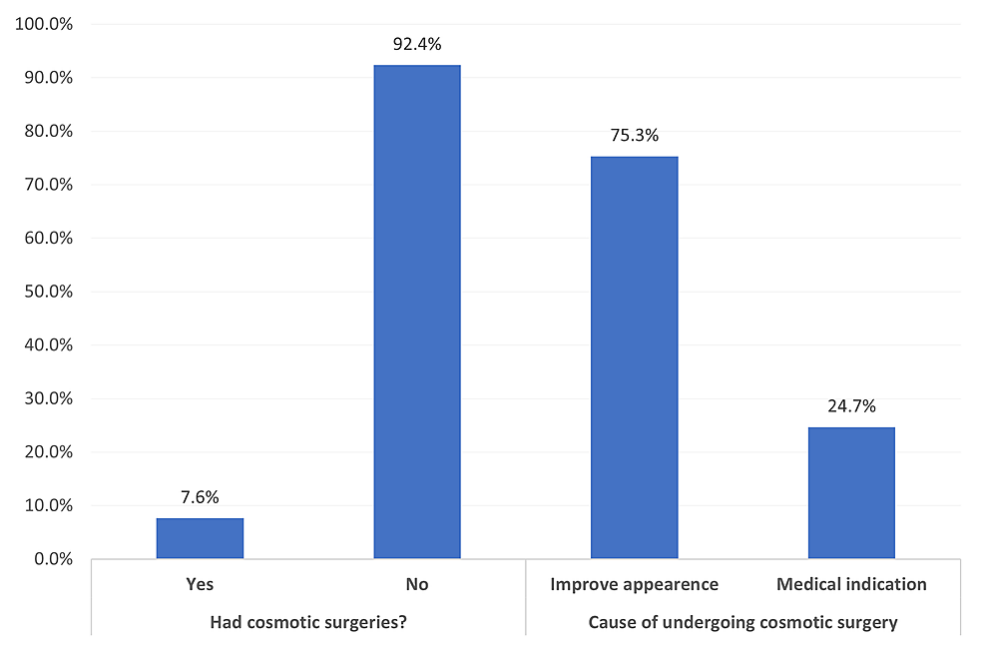
History of cosmetic surgery and related causes among the participants

Figure 1 shows the history of cosmetic surgery and related causes among the population in the eastern region, Saudi Arabia. The exact 77 (7.6%) respondents reported undergoing cosmetic surgery. Among those who had undergone the surgery, improving appearance was reported by 58 (75.3%) while 19 (24.7%) reported undergoing the surgery for a medical indication.

Table 2 illustrates descriptive of acceptance of cosmetic surgery, body acceptance, and self-esteem among study participants. As for acceptance of cosmetic surgery, the study participants showed an average level of acceptance with a mean score % of 55.4% (58.2 out of 105 points). As for body appreciation, it was high among the study respondents where the average score % was 74.2% (48.2 out of 65 points). The study participants showed more than the average level of self-esteem where they reported 24.7 out of 40 points for self-esteem with a mean score % of 61.8%.

**Table 2:** Descriptive of acceptance of cosmetic surgery, body acceptance, and self-esteem among study participants

Scale	Range	Mean	SD	Mean %
Acceptance of Cosmetic Surgery Scale (15–105)	15–105	58.2	20.5	55.4%
Body Appreciation Scale (13–65)	13–65	48.2	12.2	74.2%
Rosenberg Self-Esteem Scale (10–40)	18–31	24.7	2.0	61.8%

Table 3 shows the distribution of participant's acceptance of cosmetic surgery by their socio-demographic data. The acceptance average score was significantly higher among old-aged participants (>45 years) than the young age group (mean score of 66.1 versus 51.9, respectively; P=0.001). Also, a mean score of 63.3 was reported for the divorced/widow group in comparison to 52.5 for single participants (P=0.001). Participants with chronic health problems had significantly higher acceptance scores than others (mean score of 62.2 vs. 57.5, respectively; P=0.008). Participants who had undergone cosmetic surgery had significantly higher acceptance scores than others (mean score of 72.6 compared to 57.1; P=0.001). Also, acceptance for cosmetic surgery was higher among those who had undergone the surgery to improve appearance than others who had undergone the surgery due to medical indications.

**Table 3:** Distribution of participants acceptance of cosmetic surgery by their sociodemographic data ^#^Independent samples t-test ^One way ANOVA *P < 0.05 (significant)

	Acceptance of Cosmetic Surgery Scale				p-value
	Minimum	Maximum	Mean	SD	
Age in years					0.001*^
18–24	15	96	51.9	16.6	
25–34	15	105	56.2	19.5	
35–45	15	105	60.6	22.2	
>45	15	103	66.1	20.7	
Gender					0.207^#^
Male	15	105	59.6	22.5	
Female	15	105	57.8	19.7	
Marital status					0.001*^
Single	15	105	52.5	18.9	
Married	15	105	59.5	20.7	
Divorced/widow	30	99	63.3	20.0	
Educational level					0.151^
Intermediate	15	87	57.5	17.2	
Secondary	15	96	55.0	21.5	
University	15	105	58.7	20.4	
Work					0.118^
Not employed	15	101	56.5	19.3	
Health care field	15	105	58.5	20.4	
Non-health care field	15	105	59.6	21.6	
Monthly income					0.089^
<5000 SR	15	99	54.7	19.8	
5000–10,000 SR	15	101	57.2	19.6	
10,000–20,000 SR	15	105	59.7	21.1	
>20,000 SR	15	101	59.2	21.1	
Have a chronic health problem					0.008*^#^
Yes	15	105	62.2	22.0	
No	15	105	57.5	20.1	
Had cosmetic surgeries?					0.001*^#^
Yes	15	103	72.6	21.0	
No	15	105	57.1	20.0	
Cause of undergoing cosmetic surgery					0.048*^#^
Improve appearance	15	103	75.2	19.4	
Medical indication	25	93	64.5	23.9	

Table 4 reveals the distribution of participant's body appreciation by their socio-demographic data. Body appreciation was significantly higher among male participants than females (mean score of 50.2 vs. 47.6, respectively; P=0.013). Also, married participants had a significantly higher level of body appreciation than single (mean score of 49.2 and 44.7, respectively; P=0.001). The highest level of body appreciation was reported among non-employed respondents than the employed group (50.1 vs. 47.3 and 47.1; P=0.001). Other factors were insignificantly associated with the participant's level of body appreciation.

**Table 4:** Distribution of participants body appreciation by their sociodemographic data ^#^Independent samples t-test ^One way ANOVA *P < 0.05 (significant)

	Body Appreciation Scale				p-value
	Minimum	Maximum	Mean	SD	
Age in years					0.125^
18–24	13	65	45.3	13.0	
25–34	13	65	50.6	11.0	
35–45	13	65	48.1	12.7	
>45	21	65	48.5	11.4	
Gender					0.013*^#^
Male	13	65	50.2	11.4	
Female	13	65	47.6	12.5	
Marital status					0.001*^
Single	13	65	44.7	13.5	
Married	13	65	49.2	11.7	
Divorced/widow	22	65	46.4	11.6	
Educational level					0.811^
Intermediate	28	64	46.3	13.1	
Secondary	22	65	48.1	12.6	
University	13	65	48.2	12.2	
Work					0.001*^
Not employed	18	65	50.1	11.9	
Health care field	13	65	47.3	12.0	
Non-health care field	13	65	47.1	12.4	
Monthly income					0.101^
<5000 SR	13	65	46.1	13.9	
5000–10,000 SR	13	65	48.8	11.7	
10,000–20,000 SR	13	65	48.7	12.2	
>20,000 SR	13	65	47.0	12.0	
Have a chronic health problem					0.703^#^
Yes	18	65	48.5	11.8	
No	13	65	48.1	12.3	
Had cosmetic surgeries?					0.764^#^
Yes	13	65	47.8	12.2	
No	13	65	48.2	12.2	
Cause of undergoing cosmetic surgery					0.256^#^
Improve appearance	13	65	48.7	11.2	
Medical indication	21	61	45.0	14.8	

Table 5 shows the distribution of participant's self-esteem by their socio-demographic data. The only significant factor that affected participant's self-esteem was their educational level where participants with lower education had a significantly higher level of self-esteem (means score of 25.6 vs. 24.0 and 24.7; P=0.009).

**Table 5:** Distribution of participants self-esteem by their sociodemographic data ^#^Independent samples t-test ^One way ANOVA *P < 0.05 (significant)

	Self-Esteem Scale				p-value
	Minimum	Maximum	Mean	SD	
Age in years					0.299^
18–24	18	29	24.5	2.0	
25–34	18	31	24.7	2.0	
35–45	18	31	24.6	2.0	
>45	18	30	24.9	2.2	
Gender					0.409^#^
Male	18	30	24.7	2.2	
Female	18	31	24.6	2.0	
Marital status					0.098^
Single	19	29	24.7	2.0	
Married	18	31	24.6	2.0	
Divorced/widow	21	29	25.3	2.3	
Educational level					0.009*^
Intermediate	23	29	25.6	1.8	
Secondary	18	29	24.0	1.8	
University	18	31	24.7	2.0	
Work					0.159^
Not employed	18	31	24.5	1.9	
Health care field	19	29	24.8	1.9	
Non-health care field	18	31	24.7	2.2	
Monthly income					0.733^
<5000 SR	20	31	24.6	2.2	
5000–10,000 SR	18	31	24.6	2.1	
10,000–20,000 SR	18	30	24.7	1.9	
>20,000 SR	19	29	24.6	1.9	
Have a chronic health problem					0.518^#^
Yes	20	31	24.8	2.1	
No	18	31	24.6	2.0	
Had cosmetic surgeries?					0.683^#^
Yes	19	28	24.6	2.2	
No	18	31	24.7	2.0	
Cause of undergoing cosmetic surgery					0.477^#^
Improve appearance	20	28	24.7	1.9	
Medical indication	19	28	24.3	2.8	

Table 6 illustrates a correlation between participant's acceptance of cosmetic surgery, their body appreciation, and self-esteem levels. There was an insignificant weak positive correlation between participants’ self-esteem and their acceptance of cosmetic surgery (r=0.052). On the other hand, participants showed a significant inverse correlation between appreciation of their bodies and acceptance of cosmetic surgery (r=−0.027; P=0.035). Also, there was a significant positive weak correlation between participant's body appreciation and their self-esteem (r=0.179; P=0.001).

**Table 6:** Correlation between participants acceptance of cosmetic surgery, their body appreciation, and self-esteem levels *P < 0.05 (significant) **P < 0.01 (highly significant)

Scale	Acceptance of Cosmetic Surgery Scale	Body Appreciation Scale	Rosenberg Self-Esteem Scale
Acceptance of Cosmetic Surgery Scale	1	−0.027*	0.052
Body Appreciation Scale		1	0.179^**^
Rosenberg Self-Esteem Scale			1

## Discussion

The main aim of conducting the current research was to assess the prevalence of cosmetic surgery among populations and also to explore the factors of cosmetic procedures affecting the eastern province in Saudi Arabia. Estimating the relationship between body image dissatisfaction and the decision to seek cosmetic surgery was also explored. Cosmetic surgery has been undergone mainly for maintenance, repair, or improvement of a person's physical appearance through surgical and medical techniques [3]. Reference to the American Society for Aesthetic Plastic Surgery that total revenue of over $9 billion was spent on aesthetic procedures in 2020 [19]. Recently, the decision to undergo plastic surgery attracted a noteworthy expanse of attention. Researches have intensive on psychosocial, evolutionary, and health behavioral factors among those who have undergone cosmetic surgery [20]. Besides, more perceptual and belief issues include quality of life, self-esteem, and body image [21]. A full assessment by Ching et al. [22] reported that a patient’s body image and perceived quality of life were the most important and reliable factors of aesthetic surgery outcomes.

The current study showed that very few percent of the study participants (about 1 out of each 12) had undergone cosmetic surgery. The main reason (three quarters) was to improve appearance while only one quarter had undergone the surgery due to medical indication. This was much lower than what was estimated by Alharethy [23] who reported that 26.2% of Saudis participants underwent a laser hair removal, 19% underwent a Botox procedure, and 14.3% underwent liposuction. Also, the authors found that most participants (31%) had a cosmetic procedure to look more beautiful which is lower than the current study reported rate.

Regarding cosmetic surgery acceptance, the current study showed that participants had an average acceptance rate for cosmetic surgery (about half with high acceptance). The acceptance rate for undergoing cosmetic surgery was significantly higher among old-aged participants, unmarried groups, and those with chronic health problems. Also, participants who had undergone the surgery reported a higher acceptance rate which means they were ready for the procedure especially those who had undergone to improve their appearance. Lunde [24] revealed that younger respondents showed higher acceptance of cosmetic surgery. This was especially the case for boys’ acceptance of social motives for obtaining cosmetic surgery in contrast to the current study findings. Although the majority of the participants of this study are female, no significance was seen between the gender for the acceptance of cosmetic surgery. South Korea [25], Iran [26], and Brazil [27] reported that females had the intention of undergoing cosmetic surgery.

Other factors behind the interest in cosmetic surgery were reported by Javo et al. [28] in Norway. These factors included dysmorphic disorder such as symptoms, body image orientation, having children, been teased for appearance, knowing someone who has had cosmetic surgery, and being advised for cosmetic surgery. Agreeableness, body image perception, education were negatively related to an interest in cosmetic surgery. Alotaibi conducted a systematic review to assess demographic and cultural differences in the acceptance of cosmetic surgery [10]. The review showed that the search for beauty through cosmetic surgery is a worldwide phenomenon, while different countries, races, and cultures differ in how readily cosmetic surgery is comprised, and in the aesthetic goals of those choosing to have it. Also, women covering crudely 90% of all cosmetic surgery patients in virtually all populations studied and consistently exhibiting greater CS knowledge and acceptance.

The current study also showed that body appreciation was very high among participants which explains the low rate of seeking cosmetic surgeries and average acceptance rate for the surgery. Irrespective of that, self-esteem was not high in acceptable rate where about one-third of the respondents had high self-esteem. There was a significant association between high self-esteem and high weight appreciation among participants. This means that one’s confidence in their body image improves their self-esteem and lowers their acceptance of cosmetic surgery. These findings were concordant with other studies that covered cosmetic surgery acceptance issues and their related factors including self-esteem and body image perception [29,30].

## Conclusions

Being aware of the reasons and motivations that make the individual considering cosmetic procedures is important for plastic surgeons for relevant outcomes in terms of psychosocial, satisfaction, and self-esteem. This is the first study in Saudi Arabia that assesses the impact of self-esteem and self-perceived on the acceptance of cosmetic surgery in the Eastern province of Saudi Arabia. We hope that our findings encourage other researchers to do more studies within different cities.

## References

[1] Breuning EE, Oikonomou D, Singh P, Rai JK, Mendonca DA (2010). Cosmetic surgery in the NHS: applying local and national guidelines. J Plast Reconstr Aesthet Surg.

[2] Swami V, Chamorro-Premuzic T, Bridges S, Furnham A (2009). Acceptance of cosmetic surgery: personality and individual difference predictors. Body Image.

[3] Swami V, Furnham A (2007). The Psychology of Physical Attraction. https://www.routledge.com/The-Psychology-of-Physical-Attraction/Swami-Furnham/p/book/9780415422512.

[4] Jovic M, Sforza M, Jovanovic M, Jovic M (2017). The acceptance of cosmetic surgery scale: confirmatory factor analyses and validation among Serbian adults. Curr Psychol.

[5] (2021). ISAPS international survey on aesthetic/cosmetic procedures in 2019. https://www.isaps.org/wp-content/uploads/2020/12/Global-Survey-2019.pdf.

[6] Li J, Li Q, Zhou B, Gao Y, Ma J, Li J (2016). Predictive factors for cosmetic surgery: a hospital-based investigation. Springerplus.

[7] Crerand CE, Franklin ME, Sarwer DB (2006). Body dysmorphic disorder and cosmetic surgery. Plast Reconstr Surg.

[8] Bidkhori M, Yaseri M, Akbari Sari A, Majdzadeh R (2021). Relationship between socioeconomic factors and incidence of cosmetic surgery in Tehran, Iran. Iran J Public Health.

[9] Farshidfar Z, Dastjerdi R, Shahabizadeh F (2013). Acceptance of cosmetic surgery: body image, self esteem and conformity. Procedia - Social and Behavioral Sciences.

[10] Alotaibi AS (2021). Demographic and cultural differences in the acceptance and pursuit of cosmetic surgery: a systematic literature review. Plast Reconstr Surg Glob Open.

[11] Al-Yahya T, AlOnayzan AH, AlAbdullah ZA, Alali KM (2020). The impact of social media engagement on body image and increased popularity toward seeking cosmetic surgery. IJMDC.

[12] Henderson-King D, Henderson-King E (2005). Acceptance of cosmetic surgery: scale development and validation. Body Image.

[13] Alsumali K, Al Shammari RA, Al Shammari SM (2018). Acceptance of cosmetic surgery among females in Princess Nora Bint Abdulrahman University. Int J Adv Res.

[14] Avalos L, Tylka TL, Wood-Barcalow N (2005). The Body Appreciation Scale: development and psychometric evaluation. Body Image.

[15] Vally Z, D'Souza CG, Habeeb H, Bensumaidea BM (2019). The factor structure and psychometric properties of an Arabic-translated version of the Body Appreciation Scale-2. Perspect Psychiatr Care.

[16] Rosenberg Rosenberg, Morris Morris (2015). Society and the Adolescent Self-Image.

[17] Piyavhatkul N, Aroonpongpaisal S, Patjanasoontorn N, Rongbutsri S, Maneeganondh S, Pimpanit W (2011). Validity and reliability of the Rosenberg Self-Esteem Scale-Thai version as compared to the Self-Esteem Visual Analog Scale. J Med Assoc Thai.

[18] Aldaqal SM, Sehlo MG (2013). Self-esteem and quality of life in adolescents with extreme obesity in Saudi Arabia: the effect of weight loss after laparoscopic sleeve gastrectomy. Gen Hosp Psychiatry.

[19] (2021). Aesthetic plastic surgery national databank statistics for 2020. https://cdn.theaestheticsociety.org/media/statistics/aestheticplasticsurgerynationaldatabank-2020stats.pdf.

[20] Schofield M, Hussain R, Loxton D, Miller Z (2002). Psychosocial and health behavioural covariates of cosmetic surgery: women's health Australia study. J Health Psychol.

[21] Ozgür F, Tuncali D, Güler Gürsu K (1998). Life satisfaction, self-esteem, and body image: a psychosocial evaluation of aesthetic and reconstructive surgery candidates. Aesthetic Plast Surg.

[22] Ching S, Thoma A, McCabe RE (2003). Measuring outcomes in aesthetic surgery: a comprehensive review of the literature. Plast Reconstr Surg.

[23] Alharethy SE (2017). Trends and demographic characteristics of Saudi cosmetic surgery patients. Saudi Med J.

[24] Lunde C (2013). Acceptance of cosmetic surgery, body appreciation, body ideal internalization, and fashion blog reading among late adolescents in Sweden. Body Image.

[25] Swami V, Hwang CS, Jung J (2012). Factor structure and correlates of the acceptance of cosmetic surgery scale among South Korean university students. Aesthet Surg J.

[26] Kasmaei P, Farhadi Hassankiade R, Karimy M, Kazemi S, Morsali F, Nasollahzadeh S (2020). Role of attitude, body image, satisfaction and socio-demographic variables in cosmetic surgeries of Iranian students. World J Plast Surg.

[27] Swami V, Campana AN, Ferreira L, Barrett S, Harris AS, Tavares Mda C (2011). The Acceptance of Cosmetic Surgery Scale: initial examination of its factor structure and correlates among Brazilian adults. Body Image.

[28] Javo IM, Sørlie T (2010). Psychosocial predictors of an interest in cosmetic surgery among young Norwegian women: a population-based study. Plast Surg Nurs.

[29] Chen J, Ishii M, Bater KL (2019). Association between the use of social media and photograph editing applications, self-esteem, and cosmetic surgery acceptance. JAMA Facial Plast Surg.

[30] Kalantar-Hormozi A, Jamali R, Atari M (2016). Interest in cosmetic surgery among Iranian women: the role of self-esteem, narcissism, and self-perceived attractiveness. Eur J Plast Surg.

